# Selenophene and thiophene-core estrogen receptor ligands that inhibit motility and development of parasitic stages of *Haemonchus contortus*

**DOI:** 10.1186/s13071-016-1612-4

**Published:** 2016-06-16

**Authors:** Sarah Preston, Junjie Luo, Yuezhou Zhang, Abdul Jabbar, Simon Crawford, Jonathan Baell, Andreas Hofmann, Min Hu, Hai-Bing Zhou, Robin B. Gasser

**Affiliations:** Faculty of Veterinary and Agricultural Sciences, The University of Melbourne, Parkville, VIC 3010 Australia; Key Laboratory of Combinatorial Biosynthesis and Drug Discovery, Wuhan University School of Pharmaceutical Sciences, Wuhan, 430072 China; Medicinal Chemistry, Monash University Institute of Pharmaceutical Sciences (MIPS), Monash University, Parkville, VIC 3052 Australia; State Key Laboratory of Agricultural Microbiology, College of Veterinary Medicine, Huazhong Agricultural University, Wuhan, Hubei 430070 China; School of Biosciences, The University of Melbourne, Parkville, VIC 3010 Australia; Structural Chemistry Program, Eskitis Institute for Drug Discovery, Griffith University, Brisbane, QLD 4111 Australia

**Keywords:** Selective estrogen receptor modulators (SERMs), Selenophene and thiophene-core SERMs, Inhibitory activity, *Haemonchus contortus*, Nematode

## Abstract

**Background:**

Parasitic worms represent a substantial disease burden in animals and humans worldwide. The control of parasitic roundworms (nematodes) relies heavily on the use of anthelmintic drugs. However, widespread drug resistance in nematodes seriously compromises the effectiveness of many anthelmintics around the world. Thus, there is a need to discover new drugs, with unique modes of action, against parasites.

**Methods:**

Here, we synthesised and tested 74 selective estrogen receptor modulators (SERMs) for in vitro*-*activity on parasitic larvae of *Haemonchus contortus* (barber’s pole worm), one of the most important nematode pathogens of small ruminants (including sheep and goats) and a key representative of one of the largest groups of parasitic nematodes (the Strongylida) of animals. We also studied the morphology of treated and untreated larvae using scanning electron microscopy (SEM), and assessed the agonistic/antagonistic activity of SERMs in a human embryonic kidney cell line using a luciferase reporter assay system.

**Results:**

We identified three SERMs (one selenophene and two thiophene-core compounds) with potent inhibitory activities (at 3–25 μM) on the motility and development of parasitic stages of *H. contortus*. An SEM examination of treated *H. contortus* revealed considerable damage to the cuticle of fourth- but not exsheathed, third-stage larvae; this damage appeared to be consistent with that observed upon treatment with monepantel but not moxidectin (control compounds).

**Conclusion:**

The potency of the three SERMs compared favourably with commercially available anthelmintics, such that they warrant further assessment as nematocides. Future studies could focus on assessing the selectivity of these SERMs to parasites, characterising their target(s) and/or designing analogs that are parasite-specific.

**Electronic supplementary material:**

The online version of this article (doi:10.1186/s13071-016-1612-4) contains supplementary material, which is available to authorized users.

## Background

Despite their major socioeconomic impact globally, parasitic worms (helminths) of animals and humans are seriously neglected, in terms of funding for the research and development of chemotherapeutics, vaccines and diagnostics. The current economic losses caused by such worms to agriculture worldwide have a substantial adverse impact on farm profitability and exacerbate the global food shortage. For example, roundworms (nematodes) of livestock cause major losses to farmers due to disease, reduced weight gain, weight loss, poor productivity (e.g. in terms of meat or milk yields) and mortality in animals. Nematodes of the order Strongylida are of paramount importance as pathogens of livestock animals, such as sheep, goats, cattle and pigs, causing gastrointestinal or respiratory diseases and associated complications. In particular, *Haemonchus contortus* (the barber’s pole worm) is a highly significant pathogen of livestock worldwide, affecting hundreds of millions of small ruminants (including sheep and goats) and causing economic losses [1, 2] estimated at tens of billions of dollars per annum. This parasite feeds on blood in the stomach (abomasum) and causes gastritis, anaemia and associated complications as well as mortality. It is transmitted orally from contaminated pasture to the host through a direct life-cycle [3]: eggs are excreted in host faeces; the first-stage larvae (L1s) develop inside eggs to then hatch (usually within one day) and develop through to the second (L2)- and third (L3)-stage larvae in about a week; infective L3s are then ingested by the host, exsheath (xL3) and, after a histotropic phase, develop through fourth (L4)-stage larvae to dioecious adults (within three weeks) in the abomasum.

The control of *H. contortus* and related nematodes has relied heavily on treatment with anthelmintic drugs, including benzimidazoles, imidazothiazole, macrocyclic lactones and amino-acetonitrile derivatives. However, the excessive use of such drugs has led or is leading to widespread resistance in these nematodes to many anthelmintics of these classes [4–11], seriously compromising their effectiveness in many regions around the world. Although a vaccine (Barbervax®, Wormvax, Albany, Australia) was released to support treatment programs against haemonchosis, there is a continual need to work toward identifying new drug targets, and developing new or repurposing existing compounds [12, 13].

In public-private and cross-disciplinary partnerships, we have developed an efficient whole-organism drug-screening assay for *H. contortus* for the discovery and subsequent repurposing of compounds to parasitic nematodes [14–16]. Interestingly, recent reports have shown that some selective estrogen receptor modulators (SERMs) have considerable activity against protistan parasites, such as *Leishmania* [17–19] and cestodes, including *Echinococcus granulosus, Taenia crassiceps* and *T. solium* [20, 21]. In these cestodes, estrogen receptor-like proteins have been identified, and the mammalian ligand (17-β-estradiol) has been shown to enhance the (asexual) reproductive rate of *T. crassiceps* [22, 23]. These studies suggest that estrogen receptors (ERs) and associated pathways exist in parasitic helminths and might represent possible anthelmintic targets.

In studies directed at human applications, in order to obtain structurally novel compounds that are relatively straight forward to synthesize or produce, we have sought to expand the chemical diversity of ligands for ERs by replacing their internal scaffolding with various heterocycles and other structurally related elements and, in the process, we have produced and evaluated a series of selenophene and thiophene-derived SERMs with selective activities on ERs (including ERα and ERβ) (Hai-Bing Zhou et al., unpublished). These findings indicated that changes to the core structure, although remote from close contact with residues in the ligand-binding pocket, can have a major impact on activity; in addition, some structural alterations can lead to super-agonism, resulting in the compound having a greater response than the endogenous ligand [24]. Although our focus has been on selective ligands for ERs of mammalian cells [25–27], we were keen to assess whether selenophene- or thiophene-core ligands, particularly if they do not have an effect on mammalian ERs, have an agonistic or super-agonistic activity on parasitic larval stages of *H. contortus*.

## Methods

### Chemistry

Unless otherwise stated, reagents and materials were obtained from commercial suppliers and were used without further purification. The tetrahydrofuran (THF) and dichloromethane (DCM) were dried over Na and CaCl_2_, respectively, and distilled prior to use. Reactions were monitored by thin layer chromatography (TLC), and column chromatography purification was performed using silica gel (230–400 mesh). NMR spectra were measured using Bruker DRX and DMX spectrometers at 400 MHz for ^1^H spectra and 100 MHz for ^13^C spectra and calibrated from residual solvent signal. In the following, the procedures for the *de novo* synthesis of selenophene- and thiophene-core compounds are described below.

#### General procedure for the synthesis of 2,5-dibromoselenophene

In the absence of light, selenophene (5.09 g, 38.9 mmol) was dissolved in dry *N*,*N*-dimethylformamide (DMF), and the solution degassed. *N*-bromosuccinimide (NBS, 2 equiv) was added in four portions within 30 min, and the orange solution then stirred at room temperature (22–24 °C) for 18 h. The reaction mixture was poured into ice water and extracted with DCM. The combined organic phases were washed with water and brine, and dried over Na_2_SO_4_. The removal of the solvent yielded 10.1 g of orange liquid, which was purified by column chromatography (silica; n-hexane); the pure product was a colourless liquid.

#### General procedure for the synthesis of 2,3,5-trisbromoselenophene

Bromine (3.1 equiv) in CHCl_3_ was added dropwise to a stirred solution of selenophene (1 equiv) in CHCl_3_ and AcOH at 0 °C over the course of 1 h. The reaction mixture was warmed to room temperature and stirred for 12 h, and then heated to 70 °C for 5 h. Upon completion of the reaction, the mixture was allowed to cool to room temperature and transferred to a large beaker. Excess bromine was evaporated at room temperature, and the resultant mixture was diluted with CHCl_3_. The organic phase was successively washed with water, diluted in aqueous NaOH solution and brine, and then concentrated. The crude crystalline product was further purified by column chromatography using hexane as an eluent to give an orange liquid.

#### General procedure for the Suzuki coupling reaction of bromoselenophene with arylboronic acid

Under Ar atmosphere, a mixture of bromoselenophene (1 equiv), arylboronic acid (3 equiv for dibromoselenophenes, 4 equiv for tribromoselenophenes), Pd catalyst (0.1 equiv), sodium carbonate (1 equiv per bromine) in an oxygen-free toluene/water (1:1) solution was stirred at 120 °C for 24 h, after which the reaction mixture was cooled to room temperature. The aqueous layer was extracted with ethyl acetate. The combined organic layers were washed with brine, dried over anhydrous MgSO_4_/Na_2_SO_4_ and then filtered and concentrated in vacuum. The product was purified by column chromatography.

#### General procedure for ether cleavage

Under Ar atmosphere, to a solution of methoxyphenyl derivative 2 (1 equiv) in dry DCM at -20 °C, boron tribromide (3 equiv per methoxy function) was added dropwise. The reaction mixture was stirred at room temperature. After 4 h, water was added to quench the reaction, and ethyl acetate was used to extract the aqueous layer. The combined organic layers were washed with brine, dried over anhydrous MgSO_4_/Na_2_SO_4_, and then filtered and concentrated in vacuum. The product was purified by column chromatography.

In total, we synthesized 52 thiophene-core analogues using the method reported by Min et al. [24] and 22 selenophene-core analogues using established procedures (Additional file 1).

### Biological assay - *H. contortus*

The selenophene and thiophene-core ligands (*n* = 74) (Additional file 1) were screened at a concentration of 20 μM on exsheathed third-stage larvae (xL3s) of *H. contortus* in 96-well microculture plates (cat. no. 3635; Corning 3650, Life Sciences, Corning, USA) using relevant control compounds (i.e. moxidectin and monepantel), as described previously [15]. In brief, compounds were dissolved to a stock concentration of 10 mM in either dimethyl sulfoxide (DMSO) or methanol (Sigma-Adrich Scientific, St. Louis, USA). These ligands were then individually diluted to the final concentration of 20 μM using Luria Bertani medium (LB) supplemented with 100 IU/ml of penicillin, 100 μg/ml of streptomycin and 2.5 μg/ml of amphotericin (LB*). Compounds were dispensed (in triplicate) into the wells of a 96-well microculture plate using a multichannel pipette. In addition, the negative controls (LB*, LB* + 0.5 % solvent; six wells each), and positive controls (20 μM of monepantel; Zolvix, Novartis Animal Health, Basel, Switzerland and 20 μM of moxidectin; Cydectin, Virbac, Carros, France; triplicate wells) and xL3s (~300/well) were dispensed into wells of the plate using an automated multichannel pipette (Viaflo Assist/II, Integra Biosciences, Zizers, Switzerland). Following a 72 h incubation at 38 °C and 10 % CO_2_, a video recording (5 s) was taken of each well of the 96-well microculture plate (containing xL3s) using a greyscale camera (Rolera Bolt CMOS, Qimaging Scientific, Surrey, Canada) and a motorised X-Y axis stage (BioPoint 2, Ludl Electronics Products, Hawthorne, USA). Individual videos were processed to calculate a motility index (MI) using an algorithm described previously [14]. MIs were normalised to the negative and positive controls (to remove plate-to-plate variation) using the program Prism (v.6 GraphPad Software, USA). Z’-scores were calculated to validate the performance of the screening assay; reliable assays achieve Z’-scores of between 0.5 and 1 [28]. A compound was recorded as having an activity if it reduced xL3 motility by ≥ 70 % following incubation for 72 h. Subsequently, the anti-xL3 activity of individual compounds was confirmed, and half maximum inhibitory concentration (IC_50_) values estimated from dose–response curves (24 h, 48 h and 72 h). Compounds that reduced the motility of xL3s were also tested for their ability to inhibit the motility of L4s (same protocol as for xL3s) and/or to affect the development of xL3s to L4s [14]. Following the measurement of L4 motility, larvae were re-incubated for 4 more days and fixed with 1 % iodine (50 μl). Then, L4 development was assessed based on the presence of a well-developed mouth/pharynx using a light microscope [14] and expressed as a percentage of the total number of larvae examined (*n* = 30).

All assays (to assess xL3 motility, L4 development and L4 motility) were performed in triplicate, three times, on separate days. Data (MI) from each assay were converted to a percentage compared with the negative control (LB* + 0.5 % solvent), and IC_50_ values determined using a variable slope four-parameter equation, constraining the top value to 100 % and using a least squares (ordinary) fit model (v.6 GraphPad Software). Significant differences in IC_50_ values were established using the extra sum-of-squares *F*-test, employing a *P*-value of 0.05 (v.6 GraphPad software).

### Scanning electron microscopy (SEM)

SEM was used to assess whether compounds that reduced motility by ≥ 70 % caused structural damage to the larval stages. The xL3s and L4s were cultivated as described previously [14, 15]. Compounds were diluted to a final concentration of 100 μM in LB*, and 50 μl were transferred to wells of a 96-well microculture plate; six wells were used for each treatment and for the negative control which contained LB* and 1 % methanol (AR1115-P2.5 L, RCI; Labscan Limited). The xL3s and L4s were resuspended in LB* at a density of ~6,000 per ml, and 50 μl were transferred into individual wells. Compounds were incubated with the larvae for 24 h at 38 °C and 10 % CO_2_. Following incubation, for each treatment, the larvae from each of the six wells were pooled into 1.5 ml Eppendorf tubes, washed 3 times in 0.9 % saline at 9,000 *g* and resuspended in 1 ml of phosphate-buffered saline (PBS). Subsequently, the larvae were fixed in 2.5 % glutaraldehyde in PBS for 2 h at room temperature, rinsed three times in PBS for 15 min, and then post-fixed in 1 % osmium tetroxide for 2 h at room temperature. Fixed larvae were rinsed again 3 times in PBS for 15 min. Then, aliquots of concentrated larvae (200 μl) were incubated on polyethyleneimine (PEI)-coated glass coverslips (pre-prepared by smearing 22 mm square glass coverslips with a 0.1 % solution of PEI and dried by heat under a cool flame). Following this incubation, the excess supernatant was removed, and the coverslips with adhered nematodes were dehydrated in increasing concentrations of ethanol (10, 30, 50, 70, 90 and 100 %) in water for 30 min. The coverslips were dried in a critical point dryer (EM CPD030, Leica, Wetzlar, Germany) and mounted on to aluminium stubs (25 mm) with double-sided carbon tabs. The coverslips were then coated with gold using a Xenosput sputter coater (Dynavac, Australia). The larvae on the coverslips were imaged using a field-emission scanning electron microscope (XL30 Philips, Netherlands) at 2.0 kV using a spot size of two; 12 representative images were taken of each sample.

### Estrogen receptor binding affinity

Relative binding affinities were determined by a competitive fluorometric binding assay, as described previously [29]. Briefly, 40 nM fluorescence tracer (coumestrol, Sigma-Aldrich) and 0.8 μM purified human ERα or ERβ ligand binding domain were diluted in 100 mM potassium phosphate buffer (pH 7.4), containing 100 μg/ml bovine γ-globulin (Sigma-Aldrich), and an equal volume of test compound was added. Incubation was for 2 h at 25 °C. The fluorescence polarisation values were measured. Binding affinities are expressed as relative binding affinity (RBA) values, with the RBA of 17-β estradiol set to 100 %. The values given are the average ± range of two independent determinations. IC_50_ values were calculated using a published formula [29].

### Gene transcriptional activity

The human embryonic kidney cell line, HEK 293 T, was maintained in Dulbecco’s Minimum Essential Medium (DMEM) (Gibco, Thermo Fisher Scientific, Scoresby, Australia) with 10 % foetal bovine serum (FBS) (HyClone, GE Healthcare Life Sciences, Pittsburgh, USA). Cells in phenol red-free DMEM with 10 % FBS were plated. HEK 293 T cells were transfected with 25 μl (per well) containing 300 ng of 3 × estrogen response element (ERE)-luciferase reporter, 100 ng of either ERα or ERβ expression vector, 125 mM calcium chloride (GuoYao, China) and 12.5 μl HEPES-buffered saline (2× HBS). The next day, the cells were treated with increasing doses of ER ligands diluted in phenol red-free DMEM with 10 % FBS. After 24 h, luciferase activity was measured using the dual-luciferase reporter assay system (Promega, Fitchburg, USA) performed according to the manufacturer’s protocol.

## Results

### Primary screening results, and half maximum inhibitory concentrations (IC_50_) values of compounds with activity on xL3 and L4 stages of *H. contortus*

**Fig. 1:**
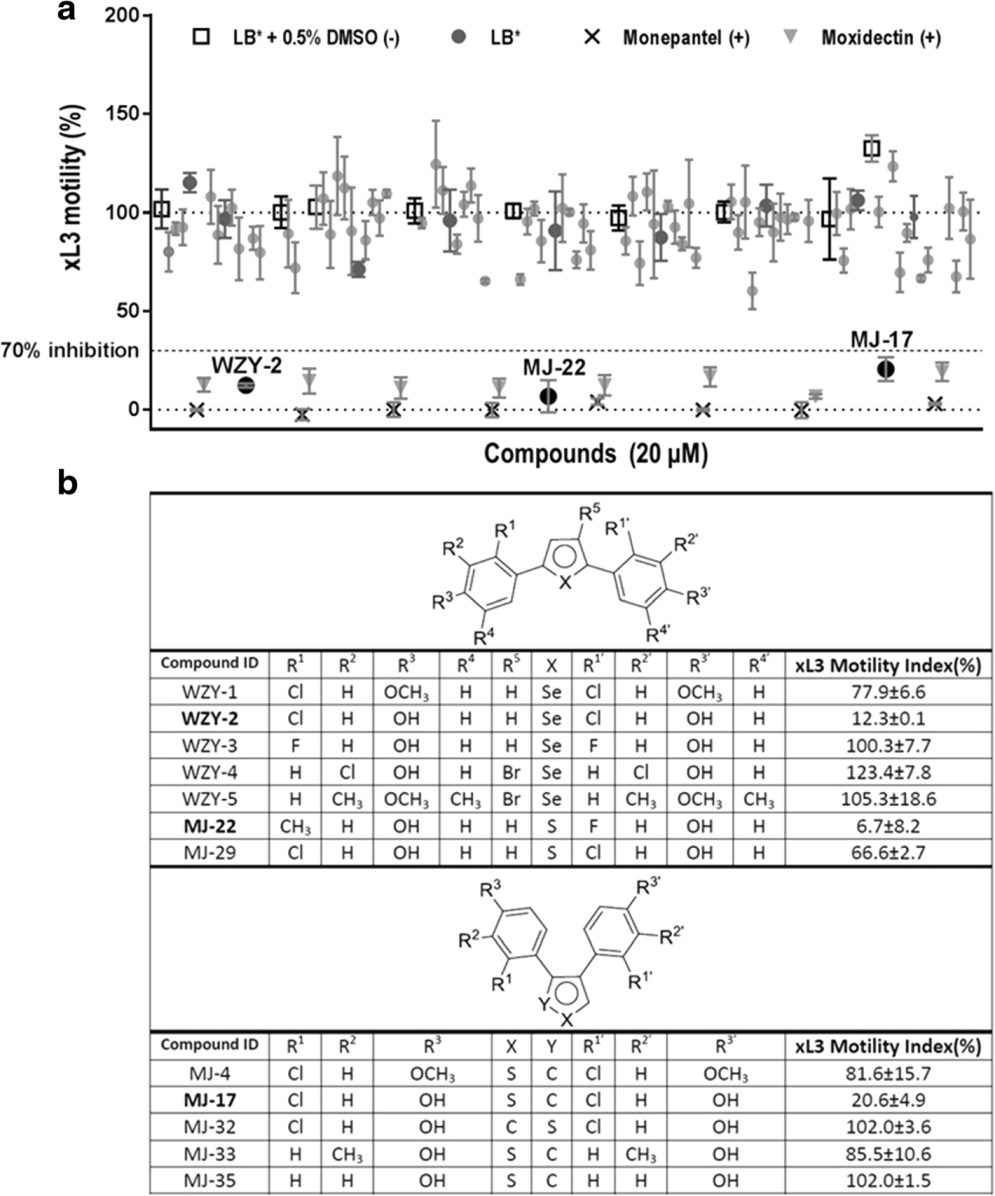
**a** Inhibitory properties of the 74 compounds at 20 μM on exsheathed third-stage larvae (xL3s) are displayed as the percent of motility compared with the positive (+) and negative (−) controls. Negative controls were xL3 treated with Luria Bertani (LB) medium supplemented with 100 IU/ml of penicillin, 100 μg/ml of streptomycin and 2.5 μg/ml of amphotericin (LB*) containing 0.5 % dimethyl sulfoxide (DMSO) or methanol (MeOH); LB* + 0.5 % DMSO/MeOH. **b** The structures and motility indices of compounds that have a similar structure to the active compounds, MJ-17, MJ-22 and WZY-2 (bold), are also shown for comparative purposes. The motility indices for individual compounds were calculated from the mean of triplicate motility indices, with the variance represented by the standard error of the mean (± SEM)

**Fig. 2:**
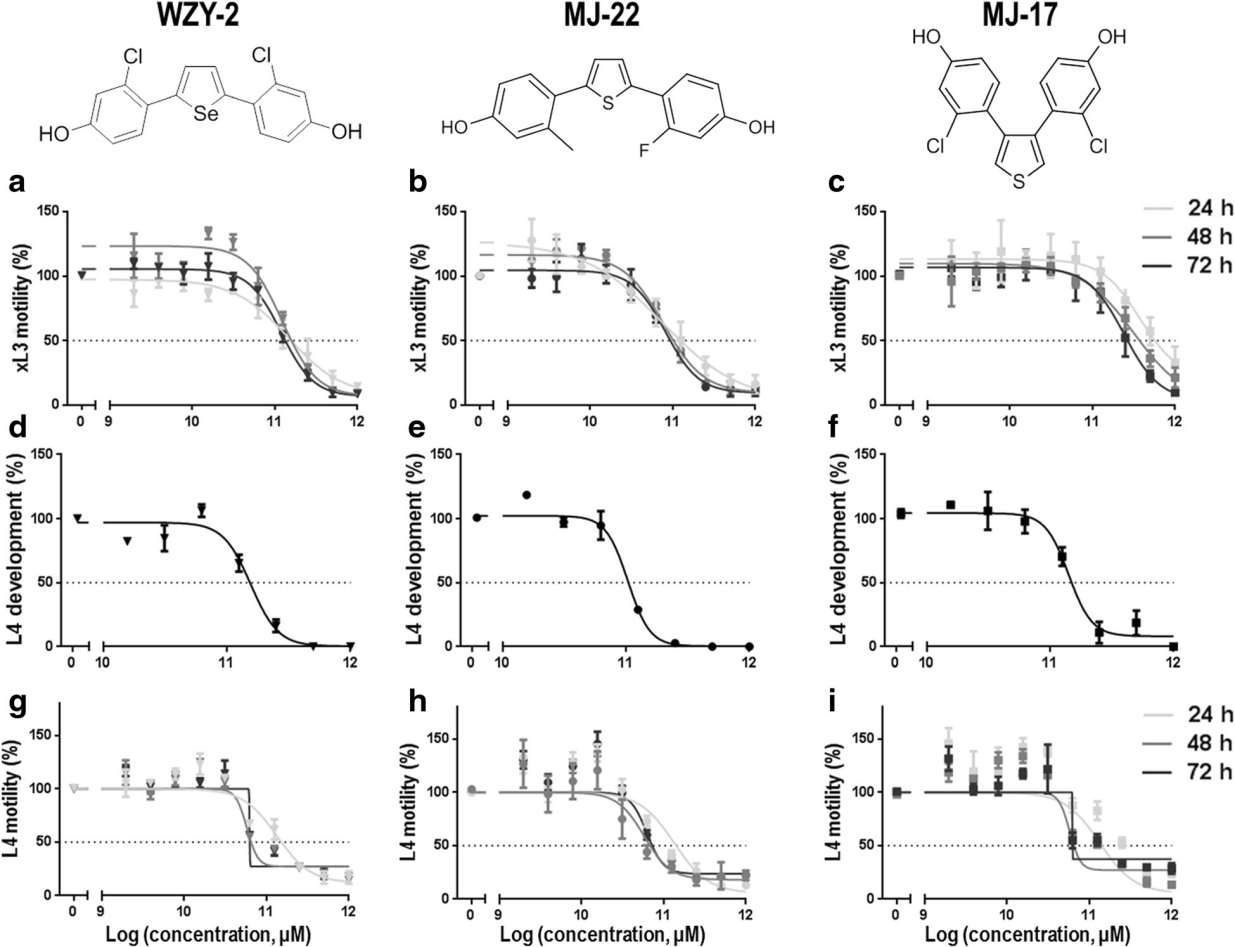
Dose–response curves for the effects of each of the selenophene-core (WZY-2) and thiophene-core (MJ-17 and MJ-22) compounds on parasitic stages of *Haemonchus contortus* in vitro. Graphs showing the inhibition of motility of third-stage larvae (xL3s) at 24 h, 48 h and 72 h (**a**-**c**) for individual compounds; inhibition of development (**d**-**f**) and motility (**g**-**i**) of fourth-stage larvae (L4s) after seven days. Each data point represents the mean of three experiments (± standard error of the mean, SEM)

In the primary screen of the 74 compounds (Fig. 1; Additional file 1), one selenophene-core compound (WZY-2) and two thiophene-core compounds (MJ-17 and MJ-22) were found to inhibit xL3 motility by > 70 %. Dose–response experiments showed that the IC_50_ values of these compounds ranged from 6.6 μM to 25.7 μM, with WZY-2 and MJ-22 being significantly more potent at inhibiting xL3 motility than MJ-17 at all three time points examined (24, 48 and 72 h) (Fig. 2; Table 1). Similar to moxidectin, all three compounds did not become significantly more potent over time, with no significant difference in the IC_50_ values of compounds at 24, 48 and 72 h (Table 1); however, although the three test compounds and moxidectin (control) had a more rapid effect on the nematode than monepantel (control), as indicated by lower IC_50_ values at the earlier time points (24 h and 48 h), monepantel was most potent at inhibiting xL3 motility at 72 h (Table 1). Subsequently, we assessed the development of xL3 to L4 in the presence of each of the three test compounds (WZY-2, MJ-22, MJ-17). All three compounds as well as moxidectin and monepantel had an inhibitory effect on L4 development, which was similar to that of moxidectin, with IC_50_ values for WZY-2, MJ-22 and MJ-17 ranging from 10.5 to 13.7 μM, and with the monepantel control compound being the most potent at inhibiting L4 development (Table 1). Compounds WZY-2, MJ-22 and MJ-17 were also found to inhibit the motility of L4s, with IC_50_ values ranging from 3.2 to 13.9 μM. Although there was no observable difference in potency between WZY-2 and MJ-22 on xL3 and L4 motility at all three time points examined, MJ-17 was significantly more potent at inhibiting the motility of L4 (IC_50_: 6.1–6.2 μM) than xL3 (IC_50_: 23.8–25.6 μM) at both 48 h and 72 h (extra sum-of-a-squares *F*-test: *F* = 5.947, *df* = 108, *P* = 0.0164; Table 1; Fig. 2).

**Table 1:** Testing of selenophene and thiophene-core ligands (WZY-2 and MJ-22, MJ-17) on *Haemonchus contortus* in the biological assay. A comparison of the ‘half of the maximum inhibitory concentration’ (IC_50_) values of individual ligands with those of the two reference anthelmintics (monepantel and moxidectin)

Compounds					
Time	WZY-2	MJ-22	MJ-17	Monepantel	Moxidectin
xL3 motility (IC_50_s in μM)					
24 h	10.0^a^	6.6^b^	25.7^a,b^	~52.1	2.5
48 h	13.0^c^	7.2^d^	25.6^c,d,g^	6.0	2.5
72 h	12.1^e^	9.6^f^	23.8^e,f,h^	0.4	2.3
L4 motility (IC_50_s in μM)					
24 h	9.3	6.4	13.9	4.3	2.2
48 h	6.1	3.2	6.2^g^	2.2	0.60
72 h	6.2	6.2	6.1^h^	3.0	0.0045
L4 development (IC_50_s in μM)					
7 days	12.2	10.5	13.7	0.4	12.3

IC_50_ values with the same superscript letter are significantly different from each other (*P* < 0.05)

### SEM of the xL3 and L4 stages of *H. contortus*

**Fig. 3:**
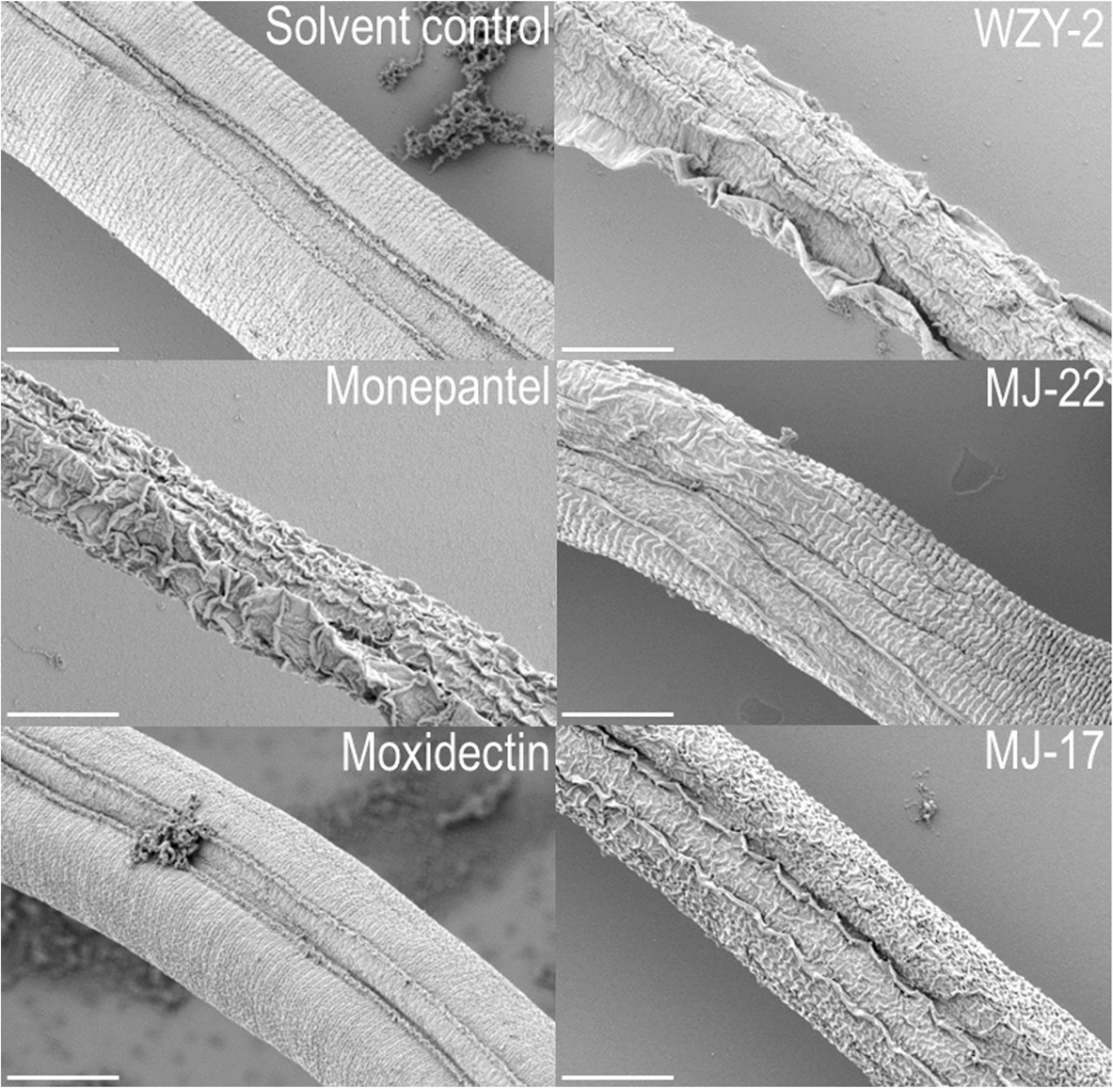
Representative scanning electron micrographs (magnification: 8,000×) of fourth-stage larvae (L4s) incubated for 24 h in either a solvent (DMSO) control, or 100 μM of each WZY-2, MJ-22, MJ-17, monepantel or moxidectin. *Scale-bars*: 5 μm

We examined by SEM the morphology of xL3 and L4 stages of *H. contortus* following incubation with 100 μM of WZY-2, MJ-22 and MJ-17 for 24 h. Although no structural damage was observed in xL3s compared with the untreated control worms, distinct damage was evident on the surface of L4s treated with each of these three compounds (Fig. 3). Compared with the surface of L4s incubated for 24 h in the solvent (DMSO) control, all three compounds disrupted the cross-sectional striations of the cuticle, induced the appearance of horizontal ridges and indentations and, in some cases, resulted in severe perturbation of the outer cuticle (Fig. 3). These features were similar to the cuticle damage observed following the treatment with 100 μM of monepantel. Moxidectin did not induce any cuticle damage in L4s at the concentrations tested, which seems consistent with a distinct mode of action (binding to ligand-gated chloride channels), resulting in influx of chloride ions and flaccid paralysis of the parasite [30].

### Transcriptional activation assays/estrogen receptor (ER) binding affinity

The binding affinities of the selenophene-core and thiophene-core ligands for both ERα and ERβ were determined by a competitive fluorometric receptor-binding assay. These affinities are presented as relative binding affinity (RBA) values, with estradiol having an affinity of 100 %. The thiophene-core compound, MJ-17, had the highest affinity to both ERs, with RBA values of 13.07 and 17.5 for ERα and ERβ, respectively, and exhibited ERβ selectivity. Compound MJ-22, which is an asymmetrically substituted compound, had lower binding affinities to these ERs (RBA values of 0.97 and 0.91 for ERα and ERβ, respectively) than corresponding symmetrical compounds. Compound WZY-2, a selenophene-core compound, had RBA values of 6.11 and 12.7 for ERα and ERβ, respectively. In the ER-responsive luciferase reporter gene assay, the ligands WZY-2 MJ-22 and MJ-17 induced transcriptional activities of genes encoding ERα and ERβ, based on comparison with 17β estradiol (E2). The luciferase assay was conducted in HEK 293 T cells transfected with the 3 × ERE-luciferase reporter. The result showed that compound WZY-2 was shown to act as an antagonist on ERβ with an IC_50_ value of 3.02 μM and a high efficacy (Table 2). Additionally MJ-17 displayed a > 25-fold higher EC_50_ value on ERα and ERβ than the corresponding 2,3-diaryl thiophenes in the micromolar range, whereas MJ-22 profiled as a super-agonist on both ERα and ERβ, being at least twice as efficacious on ERβ than estradiol (Table 2).

**Table 2:** Effects of selenophene- and thiophene-core ligand conjugates on the transcriptional activities of estrogen receptors α and β

	Agonist mode^a^				Antagonist mode^b^			
	ERα		ERβ		ERα		ERβ	
Cmpd	EC_50_ (μM)	Efficacy (%E_2_)	EC_50_ (μM)	Efficacy (%E_2_)	IC_50_ (μM)	Efficacy (%E_2_)^c^	IC_50_ (μM)	Eff (%E_2_)
WZY-2	–	-21 ± 13	–	–	–	73 ± 8	3.02	13.9 ± 5.3
MJ-22	0.47	117 ± 8	0.61	277 ± 15	–	–	–	–
MJ-17	0.045	90 ± 7	0.203	57 ± 3	–	–	–	–

^a^Luciferase activity was measured in HEK 293 T cells transfected with 3 × estrogen response element (ERE)-driven luciferase reporter and expression vectors encoding estrogen receptor α (ERα) or ERβ and treated in triplicate with increasing doses (up to 10^-5^ M) of the compounds. Half maximal effective concentrations (EC_50_) and standard deviation (mean ± SD), shown as a percentage of 10^-8^ M 17β-estradiol (E_2_), were determined

^b^Half maximal inhibitory concentrations (IC_50_) and standard deviation (mean ± SD) were determined in the percentage of 10^-8^ M 17β-estradiol (E_2_) on ERα or ERβ

^c^ERs have considerable basal activity in HEK 293 T cells. Omitted EC_50_ or IC_50_ values were too high to be determined accurately

## Discussion

Infections and diseases caused by parasitic worms impose a substantial economic burden to the livestock production industry worldwide, and cause a loss of > 5 million disability-adjusted life years (DALYs) in humans [2, 31, 32]. The control of parasitic nematodes of animals relies heavily on the use of chemical treatments [33]. Consequently, the identification of new anthelmintics is pivotal to circumvent the rapid spread and widespread occurrence of drug resistance in parasitic nematodes populations, particularly of small ruminants [4–11]. In an effort to work toward identifying possible new candidates, we screened, using a recently developed and validated in vitro-screening platform [14, 15], a compound library containing 74 compounds, and identified three hetero-cyclic core SERMs, designated WZY-2, MJ-22 and MJ-17, which have a potent inhibitory effect on both the motility and development as well as the structural integrity of parasitic larvae of *H. contortus* in vitro.

SERMs are defined as molecules that act as antagonist or agonist upon binding to ERs [34]. There are two well-known ER subtypes in humans, ERα and ERβ, which are proposed to contribute to the high selectivity and target-site specificity of SERMs [34–37]. For instance, the SERM tamoxifen, used to treat breast cancer, acts as an antagonist of the ERα in breast parenchyma. However, in the endometrial and skeletal tissues, this SERM acts as an agonist on the ERβ, and can increase the risk of uterine cancer [34, 38]. The three SERMs identified in this screen are structurally quite distinct from tamoxifen. MJ-17 and MJ-22 are thiophene-core compounds that act as agonists on human receptors ERα and ERβ. By contrast, WZY-2, a selenophene-core compound that differs from MJ-17 at only one position on the pentane ring (selenium instead of sulphur), acts as an antagonist on the ERβ receptor (Table 1; Fig. 1).

Of the 74 compounds screened, nine SERMs had structural similarity to WZY-2, MJ-22 and MJ-17, but did not have any effect on the motility of xL3s of *H. contortus*. The structures of these SERMs and their ability to reduce xL3 motility at 20 μM are shown in Fig. 1. Since selenium is, in many respects, similar to sulfur in its properties, from a medicinal chemistry perspective, two structural activity relationship (SAR) classes can be categorized, depending on the position of diphenyl substitution on the heteroaryl ring, being either a 2,5-substitution to give more “linear” molecules, such as WZY-2 and MJ-22, or a 3,4-substitution to provide a more “compact” system, such as is the case for MJ-17.

Considering first the 2,5-diphenyl substituted series, as exemplified by selenophene WZY-2 and thiophene MJ-22, replacing the chloro substituents in WZY-2 with fluoro substituents results in WZY-3, with a greatly increased xL3 motility index of 100 %. Thus, the chloro groups are highly favourable. Since WZY-3 is already very hydrophobic, with a CLogP of 5.5, the improvement in activity is unlikely to relate to the hydrophobicity-induced improved transport to the parasite, but rather an occupation of local hydrophobic binding pockets. The ortho substitution will cause the biaryl system to be non-planar, but this conformation should be readily achievable in WZY-3, such that conformational preferences are unlikely to be the cause of the differential biological activities. When the selenium atom of WZY-2 is replaced with a sulfur atom, to give MJ-29, there is a decrease in inhibitory activity, with an xL3 motility index of 67 %. Further, the potent activity of MJ-22 (xL3 motility index: 6.7 %; Fig. 1) is quite remarkable, since it possesses phenolic substituents (methyl and fluoro) that would be expected, on the basis of the above observations for WZY-2 and WZY-3, to be significantly less active. These two observations are suggestive of an SAR sub-stream for 2,5-diphenylthiophenes that is different from 2,5-diphenylselenophenes, perhaps as a result of the smaller bond lengths involved with sulfur compared with selenium, causing a less linear overall topography. Little more can be added to the discussion at this stage, without an analysis of a more extensive set of 2,5-diphenylthiophenes. That at least one of the phenolic hydroxy groups of WZY-2 is important for activity is witnessed by the limited reduction in motility of *H. contortus* caused by WZY-1 (Fig. 1), the direct analogue of WZY-2, for which both phenolic hydroxyl groups have been methylated. WZY-4 and WZY-5 have both “lost” activity, but multiple substituent changes in the phenyl and selenophene rings make it challenging to explain the cause of this loss of activity and to establish whether it is due to the bromination of the selenophene or the altered substitutions in the phenyl rings.

An analysis of the 3,4-diphenyl-substituted thiophene series, as exemplified by MJ-17, immediately reveals that the position of the sulfur atom is crucial for good activity, since MJ-32, with the sulfur atom effectively rotated one place anti-clockwise in this resultant 3,4-diphenylthiophene, has lost all activity. As one would not expect a large conformational effect on these rigid scaffolds, this result suggests that the sulfur atom in MJ-17 plays a key role in the binding interaction with the host target, possibly through the ability of sulfur to polarize, to a great degree, in order to “favourably adapt” to a protein’s “local environment” [39, 40]. That the chloro groups assume an important role is illustrated by the fact that their absence from MJ-35 results in a complete loss of activity. As the chloro groups are in ortho positions, an obvious influence on biological activity could be the direction of a favourable binding conformation, but this could be combined with a specific hydrophobic interaction with the target protein (ER). It seems unlikely that these groups contribute to transport to the site, as MJ-35 is already relatively hydrophobic, with a CLogP of 4.7. Poor activity for MJ-4 reveals the importance of at least one hydroxyl group for better activity, while similarly poor activity for MJ-33 would be consistent with a suboptimal engagement of an ortho-chloro binding pocket.

Potent activity in two topographically distinct series can often suggest non-specific modes of action and, indeed, polyphenols are known to non-specifically alter membranes at micromolar concentrations [41]. However, poor activity for analogs, such as WZY-3, MJ-29 and WZY-4 of the 2,5-substituted series, and MJ-32 and MJ-35 of the 3,4-disubstitued series is reassuring, and suggests that the activity of MJ-17 is specific. Furthermore, ERs in mammalian cell lines have been shown to bind these two types of topographically distinct series, and a similar SAR is observed (Table 2.). This information is supportive of a specific SERM mode of action, but also raises the challenge of obtaining divergent SAR between host and parasite ERs.

Previously, SERMs, such as tamoxifen and raloxifene, have been shown to reduce the burden of parasitic cestodes in experimental infections in hamsters or mice [18, 20, 21, 23]. In particular, tamoxifen is believed to interfere with the ER pathway in the parasite, as high levels of estrogen have been shown to increase the viability and reproductive rate of the asexual (cysticercus) stage of *T. crassiceps*, and treatment with this drug was shown to reduce the production of ER-like proteins [22, 23]. It is possible that the SERMs studied here (i.e. WZY-2, MJ-17 and MJ-22) might block a similar pathway, leading to reduced motility/viability as well as fertility in the ensuing *H. contortus* adults, an aspect warranting future study.

Thiophene compounds, similar to MJ-22 and MJ-17, but differing mainly by the loss of one of the phenyl groups have been associated with anti-parasitic activity in worms, such as *H. contortus* and the closely related species, *Trichostrongylus colubriformis* [42]. Gonzalez et al. [42] reported a series of thiophene analogues with potent in vitro activity, inhibiting the motility of ensheathed L3s of *H. contortus* and *T. colubriformis.* However, activity against these two nematode species was not detected *in vivo* in infected gerbils. This lack of in vivo activity of these thiophene compounds was hypothesized to relate to differences in how the drugs interact with free-living L3s in vitro compared with parasitic L4s and adults in vivo [42]. An advantage of the present screening platform [14, 15] is that compounds can be tested in vitro for activity on the parasitic (i.e. xL3s and L4s) rather than free-living stages, overcoming possible variation in efficacy linked to free-living stage usually tested in vitro (i.e. L1-L3) *vis-à-vis* the parasitic stages in vivo [42].

While WZY-2, MJ-17 and MJ-22 were initially identified in a primary screen against the xL3 stage, all compounds had inhibitory activity on L4s. WZY-2 and MJ-22 were found to be significantly more potent than MJ-17 at impeding xL3 motility, suggesting a clear delineation in efficacy between the “linear” SERM series and “compact” SERM series. However, all compounds were found to have a similar potency at inhibiting both motility and development of the L4 stage. When comparing the activity of the compounds on the two parasitic larval stages (xL3 and L4), MJ-17 was found to be more potent at inhibiting the motility of L4s than of xL3s. However, this was not the case for WZY-2 and MJ-22, which were shown to have a similar inhibitory activity on both of these larval stages. This differential efficacy against these stages was reflected in scanning electron micrographs, showing distinctiveness in the phenotypes of the two larval stages following incubation with each of the three SERMs (WZY-2, MJ-22 and MJ-17) for 24 h. While no adverse effect of individual compounds was observed on the morphology of xL3s, despite an inhibition of their motility, treated L4s were severely damaged, to a similar extent to that observed using the equivalent dose of monepantel (Fig. 3). The differences in the phenotype between xL3s and L4s, despite the same loss in motility at the same concentration, could relate to a number of reasons, such as a differing magnitude of drug uptake by the parasite, binding to the target(s) and accumulation therein. Indeed, a key morphological difference between xL3 and L4s is the presence of a prominent mouth/pharynx in the L4s compared with xL3s [43, 44]. Therefore, drug uptake (via ingestion) might be considerably higher in L4s through active feeding [44] than in xL3s, in which absorption/diffusion could be mainly trans-cuticular. Moreover, it is possible that the drug target(s), presently proposed to be ERs, might be expressed at a higher level in the L4 stage, allowing greater binding and accumulation of the compounds, thus effecting reduced motility and pronounced morphological alterations in this developmental stage.

## Conclusion

This study has identified three SERMs with potent anti-nematode activity on parasitic larvae of *H. contortus.* These SERMs might have potential as drugs against this and related nematodes, provided that analogs can be designed that act specifically on the nematode and not the host animal. Further work needs to confirm that the target(s) of these SERMs in *H. contortus* are indeed ERs and explore their structures. The design of nematode-specific SERMs, as probes, would also allow for explorations of ERs and associated pathways in *H. contortus* and related parasites.

## Additional file

Additional file 1: Details of the synthetic routes for the 22 selenophene-core and 52 thiophene-core analogues screened against *Haemonchus contortus* in the present study. The characteristics of these compounds are also given. (DOC 2062 kb)
