# Supplementary material for: Selenophene and thiophene-core estrogen receptor ligands that inhibit motility and development of parasitic stages of Haemonchus contortus
Source: Parasit Vectors. 2016 Jun 16;9:346. doi: 10.1186/s13071-016-1612-4 (PMC4910235; doi:10.1186/s13071-016-1612-4)
Supplement: Additional file 1: — Details of the synthetic routes for the 22 selenophene-core and 52 thiophene-core analogues screened against Haemonchus contortus in the present study. The characteristics of these compounds are also given. (DOC 2062 kb) [file 13071_2016_1612_MOESM1_ESM.doc]

**Supporting Information for**

Discovery of selenophene and thiophene-core estrogen receptor ligands that inhibit motility and development of *Haemonchus contortus*

Sarah Preston^a^, Junjie Luo^b^, Yuezhou Zhang^a,f^,, Abdul Jabbar^a^, Simon Crawford^e^, Jonathan Baell^f^, Andreas Hofmann^a,d^, Min Hu^c^, Hai-Bing Zhou^b^, Robin B. Gasser^a,*^

^a^ *Faculty of Veterinary and Agricultural Sciences, The University of Melbourne, Parkville, Victoria 3010, Australia*

^b^ *Key Laboratory of Combinatorial Biosynthesis and Drug Discovery (Wuhan University), Ministry of Education, State Key Laboratory of Virology, Wuhan University School of Pharmaceutical Sciences, Wuhan 430072, China*

^c^ *State Key Laboratory of Agricultural Microbiology, College of Veterinary Medicine, Huazhong Agricultural University, Wuhan 430070, Hubei, China*

^d^ *Structural Chemistry Program, Eskitis Institute for Drug Discovery, Griffith University, Brisbane, Queensland 4111, Australia*

^e^ *School of Biosciences, The University of Melbourne, Parkville, Victoria 3010, Australia*

^f^ *Medicinal Chemistry, Monash University Institute of Pharmaceutical Sciences (MIPS), Monash University, Parkville, Victoria 3052, Australia*

**Content**

[Part 1. Synthetic route for HX 3](#__RefHeading___Toc436384574)

[1.2. Synthetic route for HX-20 3](#__RefHeading___Toc436384575)

[1.3. Synthetic route for HX-46 3](#__RefHeading___Toc436384576)

[Part 2. Synthetic route for LY-1~9 4](#__RefHeading___Toc436384577)

[Part 3. Synthetic route for wzy-1~5 5](#__RefHeading___Toc436384578)

[Part 4. Synthetic route for YYS-19 8](#__RefHeading___Toc436384579)

[Part 5. Synthetic route for MJ-1~41 9](#__RefHeading___Toc436384580)

[Part 6. Synthetic route for YL-1~9 15](#__RefHeading___Toc436384581)

[Part 7. Synthetic route for WZY2-1~ WZY2-3 17](#__RefHeading___Toc436384582)

[Part 8. Synthetic route for LJJ-1~8 18](#__RefHeading___Toc436384583)

# Part 1. Synthetic route for HX

## 1.1. Synthetic route for HX-20

***General procedure for the Michael addition of β-ketosulfones to nitroalkenes:***

In an ordinary vial, the corresponding catalyst **L1** (12.8 mg, 0.01 mmol) was added to a stirred solution of nitroalkene (0.4 mmol) and *β*-ketosulfone (0.2 mmol) in THF (1.0 mL) at room temperature. After 24h, the reaction was completed (monitored by TLC analysis), the crude product was directly purified by flash column chromatography to afford product.

***General procedure for the synthesis of nitrones:***

Zn (450 mg) and saturated NH_4_Cl (7.0 mL) were added at rt to the flask equipped with a stirring bar charged with sulfone (0.1 mmol) and dry THF (7.0 mL). After 1.5h, the reaction was completed (monitored by TLC). Then the crude reaction mixture was filtered through celite and washed with dichloromethane. The organic phase was separated and the aqueous phase was extracted with CH_2_Cl_2_. The solvent was removed under reduced pressure, and the residue was purified by column chromatography (ethyl acetate: petroleum ether = 1 : 2) to give nitrone.[^1^](#_ENREF_4)

## 1.2. Synthetic route for HX-46

***General procedure for the synthesis of hydrazides:***

To a solution of (2,6-dichlorophenyl)hydrazine hydrochloride (55.5 mg, 0.26 mmol) in 3 mL of CH_2_Cl_2_ was added 52.6 mg (2.6 eq.) of Et_3_N. The mixture was stirred at room temperature for 0.5 h and then added the 4-(tert-butyl)benzoyl chloride (39.3 mg, 0.2 mmol) at 0 ^o^C. After the reaction was completed, removing the solvents, and the residue was purified by flash chromatography on silica gel, eluting with petroleum ether: ethyl acetate (8:1) to yield the product.

| Entry | Compound | Structure | Data |
| --- | --- | --- | --- |
| 1 | HX-44 |  | 87% yield. ^1^H NMR (400 MHz, CDCl_3_) *δ* 8.06 (s, 1H), 7.80 (s, 1H), 7.49 (d, *J* = 7.6 Hz, 1H), 7.30 (s, 1H), 7.09 (d, *J* = 8.2 Hz, 1H), 6.89 (d, *J* = 8.5 Hz, 1H), 6.60 (s, 1H), 1.35 (s, 9H). ^13^C NMR (101 MHz, CDCl_3_) *δ* 167.46, 156.32, 142.93, 129.17, 128.84, 127.71, 127.06, 125.86, 125.71, 120.24, 114.57, 35.09, 31.12. |
| 2 | HX-54 |  | 90% yield. ^1^H NMR (400 MHz, Acetone-*d_6_*) *δ* 9.75 (s, 1H), 7.93 (d, *J* = 8.1 Hz, 2H), 7.50 (s, 1H), 7.35 – 7.31 (m, 2H), 7.16 (d, *J* = 8.0 Hz, 1H), 6.95 (s, 1H), 6.88 (d, *J* = 8.2 Hz, 1H), 6.77 (dd, *J* = 7.8, 1.1 Hz, 1H), 2.69 (q, *J* = 7.6 Hz, 2H), 1.23 (t, *J* = 7.6 Hz, 3H). ^13^C NMR (101 MHz, Acetone-*d_6_*) *δ* 167.67, 152.37, 149.40, 147.81, 135.12, 134.46, 131.15, 130.84, 129.99, 128.89, 128.44, 128.24, 126.85, 125.24, 120.02, 113.51, 112.45, 29.37, 15.86. |
| 3 | HX-56 |  | ^1^H NMR (400 MHz, Acetone-*d_6_*) *δ* 9.68 (s, 1H), 7.79 (d, *J* = 7.9 Hz, 2H), 7.18 (dd, *J* = 14.5, 8.0 Hz, 3H), 6.97 (dt, *J* = 22.0, 7.8 Hz, 3H), 6.67 (t, *J* = 7.2 Hz, 1H), 2.55 (dd, *J* = 15.1, 7.5 Hz, 2H), 1.09 (t, *J* = 7.6 Hz, 3H). ^13^C NMR (101 MHz, Acetone-*d_6_*) *δ* 167.57, 149.46, 146.21, 131.21, 130.04, 128.89, 128.64, 128.46, 121.30, 119.48, 114.59, 29.38, 15.85. |
| 4 | HX-57 |  | ^1^H NMR (400 MHz, Acetone-*d_6_*) *δ* 9.54 (s, 1H), 7.78 (d, *J* = 7.9 Hz, 2H), 7.21 (d, *J* = 8.1 Hz, 2H), 7.06 (s, 1H), 6.82 (d, *J* = 6.5 Hz, 4H), 2.57 (q, *J* = 7.6 Hz, 2H), 1.10 (t, *J* = 7.6 Hz, 3H). ^13^C NMR (101 MHz, Acetone-*d_6_*) *δ* 167.55, 159.12, 156.79, 149.25, 147.21, 131.54, 128.83, 128.34, 116.07, 115.85, 115.36, 115.28, 29.33, 15.83. |

**Part 2. Synthetic route for LY-1~9**

***General procedure to synthesize the*** ***Pyrazolo-isoindole compounds LY-1~9:***

The mixture of *o*-alkynylchalcone **1a** (0.2 mmol), hydrazinedihydrochloride (1.0 mmol, 5 equiv), and TEA (2.0 mmol, 10 equiv) in methanol (2 mL) was refluxed for 12 h. Then, 5 mL of water was added, extracted with ethyl acetate (3×20 mL). Then the organic layer was dried with anhydrous sodium sulfate. Evaporation of ethyl acetate gave a yellow residue, which was further purified by gel column chromatography to afford the pure product.^2^

# Part 3. Synthetic route for wzy-1~5

Reagents and conditions: (a) DMF; (b) [Pd] catalyst, Na_2_CO_3_, toluene/water (1:1), reflux, 24 h; (c) BBr_3_, CH_2_Cl_2_, −20 °C to rt, 4 h.

Reagents and conditions: (a) CHCl_3_, AcOH, Br_2_; (b) [Pd] catalyst, Na_2_CO_3_, toluene/water (1:1), reflux, 24 h; (c) BBr_3_, CH_2_Cl_2_, −20 °C to rt, 4 h.

Reagents and conditions: (a) CHCl_3_, Br_2_, AcOH, rt, 18h; (b) [Pd] catalyst, Na_2_CO_3_, toluene/water (1:1), reflux, 24 h; (c) BBr_3_, CH_2_Cl_2_, −20 °C to rt, 4 h

**EXPERIMENTAL SECTION:**

***General Procedure for synthesis of 2,5-dibromoselenophene 1****.*

In the absence of light, selenophene (5.09 g, 38.9 mmol) was dissolved in dry *N*,*N*-Dimethylformamide (DMF), and the solution degassed. *N*-Bromosuccinimide (NBS, 2 equiv) was added in four portions within 30 min, and the orange solution then stirred at room temperature (22-24 °C; rt) for 18 h. The reaction mixture was poured into ice-water and extracted with dichloromethane (DCM). The combined organic phases were washed with brine and water, and dried over Na_2_SO_4_. The removal of the solvent yielded 10.1 g of orange liquid, which was purified by column chromatography (silica; *n*-hexane). The pure product was obtained as a colourless liquid.

***General procedure for 2,3,5-Trisbromoselenophene 4.***

Bromine (1 equiv) in CHCl_3_ was added dropwise to a stirred solution of selenophene (10equiv) in CHCl_3_ and AcOH at 0 °C over the course of 1 h. The reaction mixture was warmed to room temperature and stirred for 12 h, and then heated to 70 °C for 5 h. Upon completion of the reaction, the mixture was allowed to cool to room temperature and transferred to a large beaker. Excess bromine was evaporated at room temperature and the resulting mixture was diluted with CHCl_3_. The organic phase was successively washed with water, dilute NaOH solution, and brine, and then concentrated. The crude crystalline product was further purified by column chromatography using hexane as an eluent to give a orange liquid.

***General procedure for Suzuki Coupling.***

Under Ar atmosphere, a mixture of bromoselenophene (1 equiv), arylboronic acid (3 equiv for disubstituted, 4 equiv for trisubstitutedselenophenes), Pd catalyst, sodium carbonate (2 equiv) in an oxygen-free toluene/water (1:1) solution was stirred at 120 °C for 24 h, after which, the reaction mixture was cooled to room temperature. The aqueous layer was extracted with ethyl acetate. The combined organic layers were washed with brine, dried over anhydrous MgSO_4_/Na_2_SO_4_ and then filtered and concentrated in vacuum. The product was purified by column chromatography (CC).

***General procedure for Ether Cleavage.***

Under argon atmosphere, to a solution of methoxyphenyl derivative (1 equiv) in dry dichloromethane at −20 °C, boron tribromide (3 equiv per methoxyl function) was added dropwise. The reaction mixture was stirred at room temperature. After 4 h, water was added to quench the reaction and ethyl acetate was used to extract the aqueous layer. The combined organic layers were washed with brine, dried over anhydrous MgSO_4_/ Na_2_SO_4_, and then filtered and concentrated in vacuum. The product was purified by column chromatography (CC).^3^

| Entry | Compound | Structure | Data |
| --- | --- | --- | --- |
| 1 | WZY-1 |  | mp 114-116 °C, ^1^H NMR (400 MHz, CD_3_OD) *δ* 7.42 – 7.39 (d, 2H), 7.36 (s, 1H), 7.10 – 7.04 (m, 4H), 6.80 – 6.77 (d, 2H), 6.71 – 6.64 (m, 4H). ^13^C NMR (101 MHz, CD_3_OD) *δ* 158.43, 157.85, 157.38, 148.47, 142.57, 141.19, 131.43, 131.35, 130.84, 129.47, 129.33, 128.81, 128.12, 116.72, 116.22, 116.12. HRMS (MALDI/DHB) calcd for C_22_H_16_O_3_Se ( M + H^+^) 407.0388 found 407.0397. |
| 2 | WZY-3 |  | mp 206-208 °C, ^1^H NMR (400 MHz, Acetone-*d_6_*) *δ* 9.18 (s, 2H), 7.63 (d, *J* = 8.9 Hz, 2H), 7.59 (d, *J* = 8.5 Hz, 2H), 6.77 (dd, *J* = 8.5, 2.4 Hz, 2H), 6.73 (dd, *J* = 13.1, 2.3 Hz, 2H). HRMS (MALDI/DHB) calcd for C_16_H_11_O_2_F_2_Se ( M + H^+^) 351.9942 found 351.9946 |
| 3 | WZY-4 |  | mp 98-100 °C, ^1^H NMR (400 MHz, Acetone-*d_6_*) *δ* 9.33 (s, 2H), 7.63 (dd, *J* = 5.7, 2.2 Hz, 2H), 7.44 – 7.39 (m, 2H), 7.08 (dd, *J* = 16.5, 8.5 Hz, 2H). ^13^C NMR (101 MHz, Acetone-*d_6_*) *δ* 156.19, 149.19, 129.12, 129.05, 125.77, 125.43, 125.20, 116.00. |
| 4 | WZY-5 |  | mp 268-270 °C, ^1^H NMR (400 MHz, Acetone-*d*_6_) *δ* 7.50 (s, 2H), 7.34 (s, 2H), 7.22 (s, 4H), 2.27 (s, 12H).^13^C NMR (101 MHz, Acetone-*d*_6_) *δ* 154.14, 149.24, 129.00, 126.74, 125.39, 125.36, 16.67. HRMS (MALDI/DHB) calcd for C_20_H_21_O_2_Se (M + H^+^) 372.0757, found 372.0761. |

# Part 4. Synthetic route for YYS-19

***General procedure for the synthesis of the product:***

With HOBT (52.7 mg, 0.39 mmol) and EDC (74.8 mg, 39 mmol) as the condensation agents, amidation reaction of aromatic acid (50.0 mg, 0.39 mmol) and arylamine (70.7 mg, 0.468 mmol) progressed in CH_3_OH at room temperature.^4^

| Compound | Structure | Data |
| --- | --- | --- |
| YYS-19 |  | 22.6% yield. ^1^H NMR (400 MHz, DMSO-*d*_6_) *δ* 8.82 (t, *J* = 5.9 Hz, 1H), 8.17 (dd, *J* = 2.9, 1.2 Hz, 1H), 7.59 (dd, *J* = 5.0, 3.0 Hz, 1H), 7.54 (dd, *J* = 5.0, 1.1 Hz, 1H), 6.87 (dd, *J* = 8.8, 4.5 Hz, 1H), 6.79 (d, *J* = 7.9 Hz, 1H), 5.99 (s, 1H), 4.36 (d, *J* = 6.0 Hz, 1H). ^13^C NMR (100 MHz, DMSO-*d*_6_) *δ* 161.91 (s), 147.19 (s), 146.01 (s), 137.65 (s), 133.53 (s), 128.74 (s), 126.74 (d, *J* = 8.5 Hz), 120.42 (s), 107.94 (d, *J* = 5.8 Hz), 100.77 (s), 42.05 (s). |

# Part 5. Synthetic route for MJ-1~41

.

ReAgents and conditions: (a) [Pd] catalyst, Na_2_CO_3_, toluene/water (1:1), reflux, 24 h; (b) BBr_3_, CH_2_Cl_2_, −20 °C to r.t, 4 h.

**EXPERIMENTAL SECTION:**

***General Procedure for Suzuki Coupling.***

Under Ar atmosphere, a mixture of bromothiophene (1 equiv), arylboronic acid (2 equiv for monosubstituted, 4 equiv for disubstituted, 5 equiv for trisubstituted thiophenes), Pd catalyst, sodium carbonate (2 equiv) in an oxygen-free toluene/water (1:1) solution was stirred at 120 °C for 24 h, after which, the reaction mixture was cooled to room temperature. The aqueous layer was extracted with ethyl acetate. The combined organic layers were washed with brine, dried over anhydrous MgSO_4_/Na_2_SO_4_ and then filtered and concentrated in vacuum. The product was purified by column chromatography (CC).

***General Procedure for Ether Cleavage.***

Under Ar atmosphere, to a solution of methoxyphenyl derivative (1 equiv) in dry dichloromethane at −20 °C, boron tribromide (3 equiv per methoxy function) was added dropwise. The reaction mixture was stirred at room temperature. After 4 h, water was added to quench the reaction and ethyl acetate was used to extract the aqueous layer. The combined organic layers were washed with brine, dried over anhydrous MgSO4/Na_2_SO_4_, and then filtered and concentrated in vacuum. The product was purified by column chromatography (CC).^4^

**EXPERIMENTAL SECTION:**

***General Procedure for Williamson reaction is following the previously reported method.^5^***

***The procedure of adding base side chain to 3,4-bis(4-hydroxyphenyl)thiophene.***

To a mixture of 3,4-bis(4-hydroxyphenyl)thiophene and 1-(2-Chloroethyl)PiPeridine hydrochloride (1.1equiv) in DMF was added K_2_CO_3_ (4equiv) and KOH (4equiv). The reaction mixture was stirred for 45 mins at 120 ^o^C in 600W microwave. To the mixture was added H_2_O and CH_2_Cl_2_. The organic layer was washed with brine, dried (Na_2_SO_4_), filtered, and concentrated under reduced pressure. The liquid was purified on a silica gel column.^6^

***The procedure of adding acid side chain to 3,4-bis(4-hydroxyphenyl)thiophene through Heck reaction***.

Under Ar atmosphere, TEA (2 mL) was added to a mixture of 3,4-diarylthiophene (1equiv), unsaturated esters (5-10 equiv), Pd catalyst, carbonate (2 equiv) in DMF, the reaction mixture was stirred at 120 °C for 24 h. After the reaction mixture was cooled to the room temperature, 2 N HCl (5 ml) was added, then extracted with EtOAc (3 × 30 mL) and washed DMF with water. The combined organic layer was washed with saturated brine, dried over Na_2_SO_4_, ﬁltered and evaporated in vacuum, and puriﬁed by column chromatography.^7^

***General Procedure for Hydrolysis.***

Esters were dissolved in methanol (4 ml) and 2 N KOH (2mL) was added. After stirred at room temperature 3 h. The reaction mixture was acidized by 2 N HCl (3mL), then extracted with EtOAc (3 × 30 mL). The combined organic layer was washed with saturated brine, dried over Na_2_SO_4_, ﬁltered and evaporated in vacuum, and puriﬁed by column chromatography.

| Compound | Structure | Data |
| --- | --- | --- |
| MJ-2 |  | ^1^H NMR (400 MHz, Acetone-*d*_6_) *δ* 8.29 (s, 1H), 7.93 (d, *J* = 15.9 Hz, 1H), 7.58 (d, *J* = 8.1 Hz, 1H), 7.52 (d, *J* = 3.3 Hz, 1H), 7.37 (d, *J* = 3.3 Hz, 1H), 7.21 (s, 1H), 7.02 (s, 2H), 6.79 (dd, *J* = 8.2, 2.1 Hz, 1H), 6.72 (d, *J* = 8.2 Hz, 1H), 6.43 (d, *J* = 15.9 Hz, 1H), 3.74 (s, 3H), 2.37 (s, 3H), 2.14 (s, 3H). |
| MJ-3/8 |  | ^1^H NMR (400 MHz, Acetone-*d*_6_) *δ* 8.85 (s, 1H), 7.44 (q, *J* = 3.2 Hz, 2H), 7.06 (d, *J* = 8.5 Hz, 1H), 6.99 (d, *J* = 8.4 Hz, 1H), 6.94 (d, *J* = 2.5 Hz, 1H), 6.84 (d, *J* = 2.5 Hz, 1H), 6.79 (dd, *J* = 8.6, 2.6 Hz, 1H), 6.69 (dd, *J* = 8.4, 2.5 Hz, 1H), 4.07 (t, *J* = 6.1 Hz, 1H), 3.97 (t, *J* = 5.9 Hz, 2H), 3.64 (s, 2H). |
| MJ-4 |  | mp 121-125 °C, ^1^H NMR (400 MHz, CDCl_3_) *δ* 7.24 (s, 2H), 6.92 (d, *J* = 8.5 Hz, 2H), 6.80 (d, *J* = 2.6 Hz, 2H), 6.59 (dd, *J* = 8.6, 2.6 Hz, 2H), 3.69 (s, 6H). ^13^C NMR (101 MHz, CDCl_3_) *δ* 159.15, 139.18, 134.06, 132.37, 127.60, 124.54, 114.66, 112.49, 55.43. |
| MJ-5  /13 |  | ^1^H NMR (400 MHz, Acetone-*d*_6_) *δ* 7.39 – 7.30 (m, 2H), 7.10 (t, *J* = 5.9 Hz, 2H), 7.03 (d, *J* = 8.5 Hz, 2H), 6.84 (d, *J* =8.7 Hz, 2H), 2.51 (t, *J* = 7.3 Hz, 2H), 2.09 - 2.06 (m, 2H). |
| MJ-7 |  | ^1^H NMR (400 MHz, CD_3_OD) *δ* 7.42 (dd, *J* = 20.0, 12.8 Hz, 3H), 6.76 (dd, *J* = 25.2, 9.1 Hz, 3H), 6.59 (t, *J* = 8.3 Hz, 1H), 4.07 (dd, *J* = 14.2, 7.1 Hz, 2H), 4.00 (dd, *J* = 8.9, 6.1 Hz, 1H), 3.76 (dd, *J* = 17.0, 4.2 Hz, 2H). ^13^C NMR (101 MHz, CD_3_OD) *δ* 165.38, 164.79, 162.93, 162.29, 157.11, 139.37, 130.37, 127.97, 125.99, 120.27, 119.19, 111.12, 110.90, 108.66, 108.45, 106.95, 106.71, 101.54, 101.28, 71.59, 71.31, 64.59, 61.59, 49.73, 49.52, 49.20, 48.77, 48.45, 37.00, 31.69, 20.93, 14.51. |
| MJ-9 |  | ^1^H NMR (400 MHz, Acetone-*d*_6_) *δ* 7.19 (d, *J* = 3.2 Hz, 1H), 7.16 (d, *J* = 3.2 Hz, 1H), 6.92 (s, 1H), 6.86 (s, 1H), 6.79 (d, *J* = 7.0 Hz, 1H), 6.67 – 6.53 (m, 3H), 4.57 (s, 2H), 2.03 (s, 3H), 1.99 (s, 3H). |
| MJ-10 |  | ^1^H NMR (400 MHz, Acetone-*d*_6_) *δ* 7.33 (q, *J* = 3.3 Hz, 2H), 7.10 (d, *J* = 8.6 Hz, 2H), 7.03 (d, *J* = 8.4 Hz, 2H), 6.81 (d, *J* = 8.7 Hz, 2H), 6.76 (d, *J* = 8.4 Hz, 2H), 3.95 (t, *J* = 6.4 Hz, 2H), 2.31 (t, *J* = 7.4 Hz, 2H), 1.75 (dd, *J* = 14.4, 6.7 Hz, 2H), 1.62 (dd, *J* = 14.8, 7.4 Hz, 2H), 1.55 – 1.37 (m, 4H). ^13^C NMR (101 MHz, Acetone-*d*_6_) *δ* 175.11, 159.19, 157.36, 142.43, 142.18, 130.94, 130.82, 130.02, 129.04, 123.99, 123.84, 115.90, 114.98, 101.27, 68.44, 34.22, 29.92, 29.63, 26.55, 25.62. |
| MJ-11 |  | ^1^H NMR (400 MHz, Acetone-*d*_6_) *δ* 7.34 (t, *J* = 3.9 Hz, 2H), 7.11 (d, *J* = 8.6 Hz, 2H), 7.02 (d, *J* = 8.4 Hz, 2H), 6.85 (d, *J* = 8.7 Hz, 2H), 6.76 (d, *J* = 8.5 Hz, 2H), 4.07 (m, 1H), 4.02 – 3.95 (m, 2H), 3.68 (m, 2H). ^13^C NMR (101 MHz, Acetone-*d*_6_) *δ* 159.08, 157.46, 142.45, 142.13, 130.91, 130.82, 130.28, 128.97, 124.03, 123.82, 115.94, 115.06, 71.41, 70.31, 64.13. |
| MJ-12 |  | ^1^H NMR (400 MHz, Acetone-*d*_6_) *δ* 7.32 (q, *J* = 3.3 Hz, 2H), 7.10 (d, *J* = 8.6 Hz, 2H), 7.03 (d, *J* = 8.5 Hz, 2H), 6.80 (d, *J* = 8.6 Hz, 2H), 6.76 (d, *J* = 8.5 Hz, 2H), 3.93 (t, *J* = 6.5 Hz, 1H), 2.28 (t, *J* = 7.4 Hz, 1H), 1.81 – 1.68 (m, 1H), 1.66 – 1.53 (m, 1H), 1.52 – 1.41 (m, 1H), 1.32 (s, 5H). ^13^C NMR (101 MHz, Acetone-*d*_6_) *δ* 175.26, 159.21, 157.35, 142.43, 142.18, 130.94, 130.82, 130.00, 129.05, 123.98, 123.83, 115.90, 114.96, 68.53, 34.32, 30.34, 30.17, 29.98, 29.79, 26.86, 25.71. |
| MJ-16 |  | ^1^H NMR (400 MHz, Acetone-*d*_6_) *δ* 8.29 (s, 1H), 7.93 (d, *J* = 15.9 Hz, 1H), 7.58 (d, *J* = 8.1 Hz, 1H), 7.52 (d, *J* = 3.3 Hz, 1H), 7.37 (d, *J* = 3.3 Hz, 1H), 7.21 (s, 1H), 7.02 (s, 2H), 6.79 (dd, *J* = 8.2, 2.1 Hz, 1H), 6.72 (d, *J* = 8.2 Hz, 1H), 6.43 (d, *J* = 15.9 Hz, 1H), 3.74 (s, 3H), 2.37 (s, 3H), 2.14 (s, 3H). ^13^C NMR (101 MHz, Acetone-*d*_6_) *δ* 206.22, 130.97, 130.19, 128.96, 116.02, 29.86. |
| MJ-18/  23 |  | ^1^H NMR (400 MHz, CDCl_3_) *δ* 7.21 (d, *J* = 3.3 Hz, 1H), 7.14 (dd, *J* = 6.0, 2.5 Hz, 2H), 7.05 (d, *J* = 8.7 Hz, 1H), 6.90 (d, *J* = 8.5 Hz, 1H), 6.62 (d, *J* = 8.5 Hz, 1H). |
| MJ-20/39 |  | ^1^H NMR (400 MHz, Acetone-*d*_6_) *δ* 7.39 – 7.30 (m, 2H), 7.10 (t, *J* = 5.9 Hz, 2H), 7.03 (d, *J* = 8.5 Hz, 2H), 6.84 (d, *J* =8.7 Hz, 2H), 2.51 (t, *J* = 7.3 Hz, 2H), 2.09 - 2.06 (m, 2H). |
| MJ-24 |  | ^1^H NMR (400 MHz, CDCl_3_) *δ* 7.21 (s, 2H), 7.06 (s, 2H), 6.89 (d, *J* = 8.4 Hz, 2H), 6.57 (d, *J* = 8.4 Hz, 2H), 4.62 (s, 4H), 4.27 (s, 4H), 2.24 (s, 6H). |
| MJ-27 |  | ^1^H NMR (400 MHz, Acetone-*d*_6_) *δ* 7.32 (q, *J* = 3.3 Hz, 2H), 7.10 (d, *J* = 8.6 Hz, 2H), 7.03 (d, *J* = 8.5 Hz, 2H), 6.80 (d, *J* = 8.6 Hz, 2H), 6.76 (d, *J* = 8.5 Hz, 2H), 3.93 (t, *J* = 6.5 Hz, 1H), 2.28 (t, *J* = 7.4 Hz, 1H), 1.81 – 1.68 (m, 1H), 1.66 – 1.53 (m, 1H), 1.52 – 1.41 (m, 1H), 1.32 (s, 5H). ^13^C NMR (101 MHz, Acetone-*d*_6_) *δ* 175.26, 159.21, 157.35, 142.43, 142.18, 130.94, 130.82, 130.00, 129.05, 123.98, 123.83, 115.90, 114.96, 68.53, 34.32, 30.34, 30.17, 29.98, 29.79, 26.86, 25.71. |
| MJ-28 |  | ^1^H NMR (400 MHz, Acetone-*d*_6_) *δ* 7.34 (t, *J* = 3.9 Hz, 2H), 7.11 (d, *J* = 8.6 Hz, 2H), 7.02 (d, *J* = 8.4 Hz, 2H), 6.85 (d, *J* = 8.7 Hz, 2H), 6.76 (d, *J* = 8.5 Hz, 2H), 4.07 (m, 1H), 4.02 – 3.95 (m, 2H), 3.68 (m, 2H). ^13^C NMR (101 MHz, Acetone-*d*_6_) *δ* 159.08, 157.46, 142.45, 142.13, 130.91, 130.82, 130.28, 128.97, 124.03, 123.82, 115.94, 115.06, 71.41, 70.31, 64.13. |
| MJ-35 |  | mp 198-201 °C, ^1^H NMR (400 MHz, Acetone-*d*_6_) *δ* 8.49 (s, 2H), 7.33 (s, 2H), 7.03 (d, *J* = 8.0 Hz, 4H), 6.75 (d, *J* = 8.0 Hz, 4H). ^13^C NMR (101 MHz, Acetone-*d*_6_) *δ* 157.36, 142.45, 130.86, 129.02, 123.64, 115.81. |
| MJ-40 |  | ^1^H NMR (400 MHz, Acetone-*d*_6_) *δ* 7.33 (q, *J* = 3.3 Hz, 2H), 7.10 (d, *J* = 8.6 Hz, 2H), 7.03 (d, *J* = 8.4 Hz, 2H), 6.81 (d, *J* = 8.7 Hz, 2H), 6.76 (d, *J* = 8.4 Hz, 2H), 3.95 (t, *J* = 6.4 Hz, 2H), 2.31 (t, *J* = 7.4 Hz, 2H), 1.75 (dd, *J* = 14.4, 6.7 Hz, 2H), 1.62 (dd, *J* = 14.8, 7.4 Hz, 2H), 1.55 – 1.37 (m, 4H). ^13^C NMR (101 MHz, Acetone-*d*_6_) *δ* 175.11, 159.19, 157.36, 142.43, 142.18, 130.94, 130.82, 130.02, 129.04, 123.99, 123.84, 115.90, 114.98, 101.27, 68.44, 34.22, 29.92, 29.63, 26.55, 25.62. |

# Part 6. Synthetic route for YL-1~9

***General Procedure for ether cleavage and Suzuki coupling is based on the previously reported work.^3^***

| Compound | Structure | Data |
| --- | --- | --- |
| YL-1 |  | 83% yield. ^1^H NMR (400 MHz, CDCl_3_) *δ* 7.45 (m, 4H), 7.32 (d, *J* = 8.1 Hz, 2H), 7.12 (t, *J* = 6.1 Hz, 3H), 6.82 (t, *J* = 8.6 Hz, 2H), 6.73 (t, *J* = 8.1 Hz, 4H), 3.72 (s, 3H), 3.69 (s, 3H). ^13^C NMR (101 MHz, CDCl_3_) *δ* 158.35, 158.22, 141.40, 139.37, 137.15, 135.53, 129.36, 128.19, 125.81, 125.64, 125.07, 124.29, 124.25, 123.71, 113.33, 113.06, 54.28, 54.18, 54.12. |
| YL-2 |  | 86% yield. ^1^H NMR (400 MHz, CDCl_3_) *δ* 7.51 – 7.42 (m, 3H), 7.36 (d, *J* = 0.9 Hz, 1H), 7.32 (d, *J* = 8.1 Hz, 2H), 7.18 – 7.08 (m, 1H), 6.70 – 6.61 (m, 2H), 6.59 (dd, *J* = 8.6, 2.4 Hz, 1H), 6.53 (dd, *J* = 11.6, 2.5 Hz, 1H), 3.75 (s, 3H), 3.72 (s, 3H). ^13^C NMR (101 MHz, CDCl_3_) *δ* 160.45, 160.10, 159.99, 159.39, 159.28, 159.16, 158.01, 157.72, 157.40, 139.26, 137.75, 137.69, 136.12, 130.52, 128.01, 127.43, 125.88, 124.28, 114.73, 113.25, 109.60, 109.31, 101.30, 101.22, 101.04, 100.96, 54.62, 54.56. |
| YL-3 |  | 77% yield. ^1^H NMR (400 MHz, CDCl_3_) *δ* 7.40 (t, *J* = 8.0 Hz,3H), 7.32 (s, 1H), 7.24 (d, *J* = 8.1 Hz, 2H), 7.16 (d, *J* = 8.5 Hz, 1H), 6.93 (d, *J* = 2.6 Hz, 1H), 6.86 (t, *J* = 3.1 Hz,1H), 6.75 (dd, *J* = 8.7, 2.6 Hz, 1H), 6.68 (dd, *J* = 8.6, 2.6 Hz, 1H), 3.71 (s, 3H), 3.70 (s, 3H). ^13^C NMR (101 MHz, CDCl_3_) *δ* 159.23, 158.57, 158.47, 138.97, 137.42, 134.88, 134.04, 132.50, 131.77, 130.74, 127.31, 127.01, 124.23, 123.90, 123.44, 123.36, 114.65, 114.19, 112.31, 112.05, 54.52, 54.46. |
| YL-4 |  | 75% yield. ^1^H NMR (400 MHz, CDCl_3_) *δ* 9.89 (s, 1H), 7.89 (d, *J* = 8.4 Hz, 2H), 7.51 – 7.35 (m, 4H), 7.16 (s, 1H), 7.15 – 7.11 (d, *J* = 4.8 Hz, 2H), 6.84 (d, *J* = 8.8 Hz, 2H), 6.76 – 6.69 (d, *J* = 8.8 Hz ,2H), 3.75 (s, 3H), 3.71 (s, 3H). ^13^C NMR (101 MHz, CDCl_3_) *δ* 190.83, 158.37, 152.06, 144.46, 142.09, 142.05, 141.53, 137.67, 135.69, 134.90, 133.62, 129.39, 128.83, 128.50, 126.97, 125.83, 125.57, 125.06, 123.59, 113.34, 113.08, 54.32, 54.22. |
| YL-5 |  | 78% yield. ^1^H NMR (400 MHz, CDCl_3_) *δ* 9.87 (s, 1H), 7.68 (d, *J* = 8.3 Hz, 2H), 7.56 – 7.49 (m, 1H), 7.45 (s, 1H), 7.32 (d, *J* = 8.2 Hz, 2H), 7.20 – 7.14 (m, 1H), 6.66 (t, *J* = 4.0 Hz, 1H), 6.62 (dt, *J* = 4.0, 1.8 Hz, 1H), 6.57 (td, *J* = 8.3, 2.5 Hz, 1H), 6.49 (dd, *J* = 10.7, 2.4 Hz, 1H), 3.83 (s, 3H), 3.35 (s, 3H). ^13^C NMR (101 MHz, CDCl_3_) *δ* 191.93, 165.04, 164.25, 162.60, 161.79, 157.86, 156.79, 143.85, 138.02, 134.49, 132.87, 132.82, 129.73, 129.10, 128.42, 126.60, 118.97, 118.69, 107.73, 107.45, 107.24, 99.88, 99.65, 55.84, 55.34. |
| YL-6 |  | 82% yield. ^1^H NMR (400 MHz, CDCl_3_) *δ* 9.90 (s, 1H), 7.72 (d, *J* = 8.2 Hz, 3H), 7.63 (s, 1H), 7.35 (t, *J* = 7.3 Hz, 3H), 7.21 (d, *J* = 8.1 Hz, 1H), 7.18 – 7.12 (m, 2H), 6.97 (s, 1H), 3.94 (s, 3H), 3.43 (s, 3H). ^13^C NMR (101 MHz, CDCl_3_) *δ* 191.85, 156.67, 156.61, 155.60, 155.57, 143.40, 138.99, 138.15, 135.38, 134.78, 132.39, 129.84, 128.45, 128.10, 117.83, 117.56, 110.00, 108.42, 108.15, 55.89, 55.32. |
| YL-7 |  | 86% yield. ^1^H NMR (400 MHz, CDCl_3_) *δ* 7.66 (s, 3H), 7.51 – 7.46 (m, 2H), 7.17 (d, *J* = 1.6 Hz, 2H), 7.14 – 7.09 (m, 2H), 6.88 – 6.84 (m, 2H), 6.79 – 6.72 (m, 2H), 3.76 (s, 3H), 3.72 (s, 3H). ^13^C NMR (101 MHz, CDCl_3_) *δ* 158.62, 158.54, 142.08, 138.13, 137.71, 133.79, 130.73, 130.40, 129.37, 127.97, 125.92, 125.38, 124.32, 122.94, 113.40, 113.28, 54.33, 54.30. |
| YL-8 |  | 84% yield. ^1^H NMR (400 MHz, CDCl_3_) *δ* 7.64 (d, *J* = 4.6 Hz, 3H), 7.51 – 7.43 (m, 1H), 7.38 (s, 1H), 7.15 (dd, *J* = 11.4, 7.1 Hz, 2H), 6.71 – 6.59 (m, 4H), 6.52 (dd, *J* = 11.7, 2.4 Hz, 1H), 3.76 (s, 3H), 3.72 (s, 3H). ^13^C NMR (101 MHz, CDCl_3_) *δ* 159.66, 158.79, 139.62, 137.37, 136.03, 135.78, 133.97, 132.41, 131.95, 130.84, 130.64, 130.31, 127.07, 126.27, 125.96, 123.69, 122.70, 114.67, 114.30, 112.40, 54.65, 54.61. |
| YL-9 |  | 79% yield. ^1^H NMR (400 MHz, CDCl_3_) *δ* 7.60 (d, *J* = 14.7 Hz, 3H), 7.45 (d, *J* = 8.6 Hz, 1H), 7.35 (s, 1H), 7.23 (d, *J* = 8.6 Hz, 1H), 7.18 (d, *J* = 3.2 Hz, 1H), 6.96 (dd, *J* = 11.6, 2.6 Hz,2H), 6.90 – 6.87 (m, 1H), 6.82 – 6.73 (m, 2H), 3.77 (s, 3H), 3.74 (s, 3H). ^13^C NMR (101 MHz, CDCl_3_) *δ* 160.54, 160.43, 160.33, 160.02, 159.57, 159.46, 157.86, 157.53, 137.76, 137.69, 136.70, 135.96, 131.51, 130.70, 130.37, 128.04, 127.14, 125.04, 123.58, 120.87, 112.89, 111.59, 109.69, 109.59, 101.29, 101.04, 54.65. |

# Part 7. Synthetic route for WZY2-1~ WZY2-3

**These compound were synthesized according to the mentioned method above.^3^**

| Compound | Structure | Data |
| --- | --- | --- |
| WZY2-1 |  | ^1^H NMR (400 MHz, CDCl_3_) *δ* 7.52-7.55 (m,4H), 6.69-6.74 (m,4H), 3.83 (s, 6H). ^13^C NMR (100 MHz, CDCl_3_) *δ* 159.4, 146.4, 132.5, 131.7, 128.9, 127.6, 115.5, 113.4, 55.6. |
| WZY2-2 |  | ^1^H NMR (400 MHz, CDCl_3_) *δ* 7.30 (s, 2H), 7.20 (s, 4H), 3.73 (s, 6H), 2.31 (s, 12H). ^13^C NMR (100 MHz, CDCl_3_) *δ* 156.8, 149.0, 132.1, 131.4, 126.5, 125.6, 59.8, 16.2. |
| WZY2-3 |  | ^1^H NMR (400 MHz, CDCl_3_) *δ* 7.64 (d, *J* = 2.2 Hz, 1H), 7.51 (d, *J* = 2.3 Hz, 1H), 7.48 (dd, *J* = 8.5, 2.2 Hz, 1H), 7.33 (dd, *J* = 8.5, 2.3 Hz, 1H), 7.30 (s, 1H), 6.97 – 6.88 (m, 2H), 3.94 (d, *J* = 2.4 Hz, 3H), 3.92 (s, 3H). ^13^C NMR (100 MHz, CDCl_3_) *δ* 155.1, 155.0, 147.2, 139.9, 130.7, 129.2, 128.6, 127.9, 127.6, 125.4, 123.2, 122.5, 112.3, 111.8, 108.4, 56.3, 56.3. |

# Part 8. Synthetic route for LJJ-1~8

ReAgents and conditions: a)Br_2_, CHCl_3_:AcOH = 6:1（v:v）, rt,12 h; 70 ^o^C,5 h; b) Zn, AcOH:H_2_O = 1:2(v:v), 37 ^o^C,12 h; c) arylboronic acid (2.2 equiv), Pd catalyst, sodium carbonate (4 equiv), oxygen-free toluene/water=1:1, 120 °C, 24 h.

***The procedure to synthesize 2,3,4,5-tetrabromsubstitued selenophene.***

Bromine (94 g) in CHCl_3_ (60 mL) was added dropwise to a stirred solution of selenophene (15 g) in CHCl_3_ (60 mL) and AcOH (10 mL) at 0 °C over the course of 1 h. The reaction mixture was warmed to room temperature and stirred for 12 h, and then heated to 70 °C for 5 h. Upon completion of the reaction, the mixture was allowed to cool to room temperature and transferred to a large beaker. Excess bromine was evaporated at room temperature and the resulting mixture was diluted with CHCl_3_ (200 mL). The organic phase was successively washed with water (80 mL), dilute NaOH solution (50 mL), and brine (60 mL), and then concentrated. The crude crystalline product was further purified by column chromatography using hexane as an eluent to give a white crystalline solid (46.5 g, 91% yield).^8^

***The procedure of debromine to synthesize 3,4-bisbromine substitued selenophene.***

2,3,4,5-Tetrabromothiophene (5.00 g, 12.6 mmol) was added to the mixture of glacial acetic acid (4 mL) and water (8 mL) followed by slow addition of zinc (4.90 g, 75.6 mmol). The reaction mixture was kept for stirring at 37 ^o^C for 12 h. The remaining zinc was filtered; the filtrate was extracted with ethyl acetate, washed with brine repeatedly. The combined organic layer was dried over Na_2_SO_4_. After removal the solvent under reduced pressure, the residue was purified by column chromatography on silica gel (petroleum ether) to give a colorless liquid.^9^

***The procedure for Suzuki coupling and ether cleavage is according to the previously reported method.***[***^14^***](#_ENREF_16)

| Entry | Compound | Structure | Data |
| --- | --- | --- | --- |
| 1 | LJJ-1 |  | ^1^H NMR (400 MHz, CD_3_Cl) *δ* 7.92 (s, 2H), 7.19 – 7.14 (m, 4H), 6.88 – 6.82 (m, 4H), 3.84 (s, 6H). ^13^C NMR (101 MHz, CD_3_Cl) *δ* 158.54, 144.02, 130.91, 130.24 , 127.87, 113.50, 55.24. |
| 2 | LJJ-2 |  | ^1^H NMR (400 MHz, CD_3_Cl) *δ* 7.96 (s, 2H), 7.20 (s, 2H), 7.05 (dd, *J* = 8.3, 2.3 Hz, 2H), 6.80 (d, *J* = 8.4 Hz, 2H), 3.90 (s, 6H), 2.32 (s, 6H). ^13^C NMR (101 MHz, CD_3_Cl) *δ* 156.78, 144.31, 131.56, 130.66, 127.67, 127.65, 126.05, 125.09, 110.26, 109.43, 55.46, 55.31. |
| 3 | LJJ-3 |  | ^1^H NMR (400 MHz, CD_3_Cl) *δ* 7.90 (s, 2H), 6.95 – 6.86 (m, 6H), 3.89 (s, 6H). ^13^C NMR (101 MHz, CD_3_Cl) *δ* 153.01, 150.57, 146.75, 146.65, 142.65, 142.64, 131.10, 131.03, 128.94, 124.94, 124.91, 116.81, 116.63, 112.93, 112.91, 56.10. |
| 4 | LJJ-4 |  | ^1^H NMR (400 MHz, CD_3_Cl) *δ* 7.67 (s, 2H), 6.84 (d, *J* = 8.4 Hz, 2H), 6.56 – 6.44 (m, 4H), 3.64 (s, 6H), 1.95 (s, 6H). ^13^C NMR (101 MHz, CD_3_Cl) *δ* 158.42, 144.47, 137.66, 131.58, 130.53, 127.78, 115.20, 110.53, 55.09. |
| 5 | LJJ-5 |  | ^1^H NMR (400 MHz, CD_3_Cl) *δ* 7.83 (s, 2H), 6.92 (d, *J* = 8.5 Hz,2H), 6.75 (d, *J* = 2.7 Hz, 2H), 6.55 (dd, *J* = 8.6, 2.6 Hz,2H), 3.64 (s, 6H). ^13^C NMR (101 MHz, CD_3_Cl) *δ* 153.97, 142.34, 131.37, 130.64, 129.03, 128.51, 121.97, 111.60, 56.12. |
| 6 | LJJ-6 |  | ^1^H NMR (400 MHz, Acetone-*d*_6_) *δ* 8.24 (s, 2H), 7.76 (s, 2H), 6.88 – 6.79 (m, 4H), 6.67 – 6.54 (m, 4H). ^13^C NMR (101 MHz, Acetone-*d*_6_) *δ* 206.68, 157.23, 144.97, 130.99, 130.84, 128.53, 115.74. |
| 7 | LJJ-7 |  | ^1^H NMR (400 MHz, Acetone-*d*_6_) *δ* 8.21 (s, 2H), 7.88 (s, 2H), 6.99 (d, *J* = 2.2 Hz, 2H), 6.81 – 6.64 (m, 4H), 2.14 (s, 6H). ^13^C NMR (101 MHz, Acetone-*d*_6_) *δ* 206.68, 155.18, 145.16, 132.32, 130.89, 128.32, 128.20, 124.54, 114.96. |
| 8 | LJJ-8 |  | ^1^H NMR (400 MHz, Acetone-*d*_6_) *δ* 8.76 (s, 2H), 8.01 (s, 2H), 6.97 – 6.81 (m, 6H). ^13^C NMR (101 MHz, Acetone-*d*_6_) *δ* 152.98, 150.59, 144.77, 144.64, 143.54 , 143.52, 131.41, 131.35, 129.91, 126.21, 126.18, 118.37, 118.34, 117.46, 117.27. |

**References**

1. Han, X.; Wu, X.; Min, C.; Zhou, H.-B.; Dong, C., An expedient approach to highly enantioenriched cyclic nitrones mediated by robust and recoverable C 3-symmetric cinchonine-squaramide catalysts. *RSC Advances* **2012,** *2* (19), 7501-7505.

2. Li, Y.; Dong, C.-E., Efficient synthesis of fused pyrazoles via simple cyclization of o-alkynylchalcones with hydrazine. *Chinese Chemical Letters* **2015,** *26* (5), 623-626.

3. Min, J.; Wang, P.; Srinivasan, S.; Nwachukwu, J. C.; Guo, P.; Huang, M.; Carlson, K. E.; Katzenellenbogen, J. A.; Nettles, K. W.; Zhou, H.-B., Thiophene-Core Estrogen Receptor Ligands Having Superagonist Activity. *Journal of medicinal chemistry* **2013,** *56* (8), 3346-3366.

4. Han, S.; Zhang, F.-F.; Qian, H.-Y.; Chen, L.-L.; Pu, J.-B.; Xie, X.; Chen, J.-Z., Development of Quinoline-2,4(1H,3H)-diones as Potent and Selective Ligands of the Cannabinoid Type 2 Receptor. *J. Med. Chem.* **2015,** *58* (15), 5751-5769.

5. Romeiro, L. A. S.; Ferreira, M. d. S.; da Silva, L. L.; Castro, H. C.; Miranda, A. L. P.; Silva, C. L. M.; Noel, F.; Nascimento, J. B.; Araujo, C. V.; Tibirica, E.; Barreiro, E. J.; Fraga, C. A. M., Discovery of LASSBio-772, a 1,3-benzodioxole N-phenylpiperazine derivative with potent alpha 1A/D-Adrenergic receptor blocking properties. *Eur. J. Med. Chem.* **2011,** *46* (7), 3000-3012.

6. (a) Patel, M. R.; Bhatt, A.; Steffen, J. D.; Chergui, A.; Murai, J.; Pommier, Y.; Pascal, J. M.; Trombetta, L. D.; Fronczek, F. R.; Talele, T. T., Discovery and Structure-Activity Relationship of Novel 2,3-Dihydrobenzofuran-7-carboxamide and 2,3-Dihydrobenzofuran-3(2H)-one-7-carboxamide Derivatives as Poly(ADP-ribose)polymerase-1 Inhibitors. *J. Med. Chem.* **2014,** *57* (13), 5579-5601; (b) Grice, C. A.; Tays, K. L.; Savall, B. M.; Wei, J.; Butler, C. R.; Axe, F. U.; Bembenek, S. D.; Fourie, A. M.; Dunford, P. J.; Lundeen, K.; Coles, F.; Xue, X.; Riley, J. P.; Williams, K. N.; Karlsson, L.; Edwards, J. P., Identification of a Potent, Selective, and Orally Active Leukotriene A4 Hydrolase Inhibitor with Anti-Inflammatory Activity. *J. Med. Chem.* **2008,** *51* (14), 4150-4169.

7. Chen, Q.; He, Y., One-pot conversion of phenols to arenes. *Synthesis* **1988,** (11), 896-7.

8. Patra, A.; Wijsboom, Y. H.; Leitus, G.; Bendikov, M., Tuning the Band Gap of Low-Band-Gap Polyselenophenes and Polythiophenes: The Effect of the Heteroatom. *Chem. Mater.* **2011,** *23* (3), 896-906.

9. (a) Ertas, E.; Ozturk, T., A new reaction of P4S10 and Lawesson's reagent; a new method for the synthesis of dithieno[3,2-b;2',3'-d]thiophenes. *Tetrahedron Lett.* **2004,** *45* (17), 3405-3407; (b) Xie, Y.; Wu, B.-M.; Xue, F.; Ng, S.-C.; Mak, T. C. W.; Hor, T. S. A., Catalytic and Stoichiometrically Directed Synthesis of Less Accessible Bromothiophenes and Bromobithiophenes. Trapping and Characterization of Catalytic Intermediates of trans-PdBr(C4H4-nBrn-1S-C)(PPh3)2 (n = 1-4), trans-PdBr(C8H4BrS2-C)(PPh3)2, and trans,trans-Pd2Br2(μ-C8H6-nBrn-2S2-C,C')(PPh3)4 (n = 2, 4). *Organometallics* **1998,** *17* (18), 3988-3995; (c) Wang, Z.; Tao, F.; Xi, L.-y.; Meng, K.-g.; Zhang, W.; Li, Y.; Jiang, Q., Two novel propylenedioxythiophene-based copolymers with donor-acceptor structures for organic solar cell materials. *J. Mater. Sci.* **2011,** *46* (11), 4005-4012.
